# Systematic review of carbapenem-resistant *Enterobacteriaceae* causing neonatal sepsis in China

**DOI:** 10.1186/s12941-019-0334-9

**Published:** 2019-11-14

**Authors:** Yijun Ding, Yajuan Wang, Yingfen Hsia, Mike Sharland, Paul T. Heath

**Affiliations:** 1 Department of Neonatology, Beijing Children’s Hospital, Capital Medical University, National Center for Children’s Health, Beijing, China; 2 Paediatric Infectious Diseases Research Group, Institute for Infection and Immunity, St. George’s University of London, London, UK; 3 Queen’s University Belfast, School of Pharmacy, 97 Lisburn Road, Belfast, BT9 7BL UK

**Keywords:** *Klebsiella pneumoniae*, *Escherichia coli*, Neonate, Genotype, Carbapenem-resistant

## Abstract

**Background:**

Carbapenems are β-lactam antibiotics which are used to treat severe infections caused by multidrug resistant *Enterobacteriacea*. The recent emergence and rapid spread of *Enterobacteriaceae* resistant to carbapenems is a global concern. We undertook a systematic review of the antibiotic susceptibility and genotypic characteristics of carbapenem-resistant *Enterobacteriaceae* in Chinese neonates.

**Methods:**

Systematic literature reviews were conducted (PubMed/Medline, Embase, Wanfang medical online databases, China National Knowledge Infrastructure (CNKI) database) regarding sepsis caused by carbapenem-resistant *Enterobacteriaceae* in Chinese neonates aged 0-30 days.

**Results:**

17 studies were identified. Eleven patients in the six studies reported the source of infection. Ten patients (10/11, 90.9%) were hospital-acquired infections. Genotypic data were available for 21 isolates in 11 studies (20 *K. pneumoniae*, 1 *E. coli*). NDM-1 was the most frequently reported carbapenem-resistant genotype (81.0%, 17/21). Carbapenem-resistant *Klebsiella pneumoniae* and *Escherichia coli* were resistant to many antibiotic classes with the exception of colistin and fosfomycin. Sequence type 105 (ST105) was the most commonly reported *K. pneumoniae* ST type (30.8%; 4/13), which was from the same hospital in Western China. ST17 and ST20 were the second and third most common *K. pneumoniae* ST type, 23.1% (3/13) and 15.4% (2/13) respectively. The three strains of ST17 are all from the same hospital in central China. The two strains of ST20, although not from the same hospital, belong to the eastern part of China.

**Conclusions:**

*Klebsiella pneumoniae* with the NDM-1 genotype was the leading cause of neonatal carbapenem resistant sepsis in China. Hospital acquired infection is the main source of carbapenem resistant sepsis. There is currently no licenced antibiotic regimen available to treat such an infection in China. Improved surveillance, controlling nosocomial infection and the rational use of antibiotics are the key factors to prevent and reduce its spread.

## Background

According to the global reports, in 2013, 51.8% of the 6.3 million children under the age of five died of infectious diseases, while 44% (276.1 million) died during the neonatal period. Neonatal sepsis is the third leading cause of neonatal death, killing 0.421 million neonates worldwide in 2013 [1]. The overall incidence of neonatal sepsis in four Asian centres (including mainland China, Thailand, Macau, and Malaysia) was 26.1 (95% CI 24.5 to 27.8) per 1000 admissions and *Klebsiella spp*. was the most common Gram negative organism causing most deaths [2]. Laxminarayan et al. [3] reported that 214 000 of 690, 000 annual neonatal deaths (31%) associated with sepsis are potentially attributable to antimicrobial resistance. Carbapenems are beta-lactam antibiotics which are used to treat severe infections caused by multidrug resistant *Enterobacteriaceae*, such as *Klebsiella pneumoniae* (*K. pneumoniae*) and *Escherichia coli* (*E. coli*). The recent emergence and rapid spread of *Enterobacteriaceae* resistant to carbapenems is therefore of global concern [4].

Resistance to carbapenems includes production of carbapenemases or a combination of structural mutations and production of other β-lactamases, such as extended-spectrum β-lactamase (ESBL) and AmpC cephalosporinases. Bacteria that produce carbapenemases, enzymes that hydrolyze carbapenems, can break down other β-lactam antibiotics including penicillins, cephalosporins, and monobactams [5]. Carbapenemases can be divided into class A (e.g. *K. pneumoniae* carbapenemase, KPC), class B metallo-β-lactamases [MBLs, e.g. New Delhi metallo-β-lactamase (NDM), Verona integrin-encoded metallo-beta-lactamases (VIM), Imipenem-resistant Pseudomonas (IMP)] and class D β-lactamases (e.g. oxacillinases OXAs). Class C β-lactamases are rarely reported [4].

Recent studies suggest that carbapenem resistance is increasing in China. A national report using data from CHINET (a Chinese antimicrobial resistance surveillance network) has shown that the overall prevalence of imipenem-resistant *K. pneumoniae* increased from 3.0% to 20.9% and meropenem-resistance from 2.9% to 24.0% between 2005 and 2017. These data included both children and adults and most of the samples were from sputum and urine. Among the five children’s hospitals, the resistance rate of *K. pneumoniae* isolated from one hospital to imipenem was 2.5%, while from the other four hospitals resistance rates ranged from 32.1% to 45.5%. Little information was available on age ranges and types of samples [6]. This systematic review aimed to summarize the current data from both English and Chinese language sources on the antibiotic susceptibility and genotypic characteristics of carbapenem-resistant *Enterobacteriaceae* (*K. pneumoniae* and *E. coli*) causing neonatal sepsis in China.

## Methods

### Definitions

Carbapenem-resistance was defined as resistance to any one of meropenem, imipenem, or ertapenem according to the US Central Laboratory Standards Institute (CLSI). In 2015 the breakpoint was changed from 2010. Laboratories using *Enterobacteriaceae* minimal inhibitory concentration (MIC) interpretive criteria for carbapenems described in M100-S20 (January 2010) performed the modified Hodge test (MHT), Carba NP test and/or a molecular assay when isolates of *Enterobacteriaceae* were suspicious for carbapenemase production based on impipenem or meropenem MICs of 2–4 ug/ml or ertapenem MIC of 2 ug/ml in 2015 [7]. Carbapenem-resistant *K. pneumoniae* or *E. coli* sepsis was defined as a laboratory confirmed culture of *K. pneumoniae* or *E. coli* obtained from the blood accompanied with signs and symptoms of infection [8]. Neonates were defined as age 0-30 days [9].

### Search strategy and selection criteria

This systematic review was conducted according to the Preferred Reporting Items for Systematic Reviews and Meta-Analyses guidance (PRISMA) [10]. We searched the published literature from PubMed/Medline, Embase, China National Knowledge Infrastructure (CNKI) and Wanfang med online databases between January 1, 2000, and June 28, 2018. We used the search terms (“beta-lactamases/or carbapenemase, or carbapenem resistance/resistant or drug resistance or carbapenemase* or carbapenem adj1 resist* or MBL or metallo-b-lactamase or VIM or NDM or OXA or oxacillinase or IMP or KPC or *Klebsiella pneumoniae* carbapenemase or OmpK”) AND (“*Enterobacteriaceae*/or *enterobacteriaceae* or *Escherichia*/or *Escherichia* or *Escherichia coli* or *Klebsiella* or *Klebsiella/Klebsiella pneumoniae/Klebsiella oxytoca*”) AND (“China or Chinese”) AND (“neonate or newborn or infant”) for English databases. We used search terms (“Carbapenems or Carbapenem”) AND (“antibiotic resistance”) AND (“infant”, OR “neonatal”) for Chinese databases. We limited the searches to Chinese territories, including Taiwan, Hong Kong, and Macau. The full search strategy is available in Additional file 1: Table S1.

### Inclusion and exclusion criteria

We include studies with original data on carbapenem-resistant *K. pneumoniae* or *E. coli* sepsis in neonates, which contained any antimicrobial resistance (AMR) or genotype data, or showed the proportion of carbapenem resistant isolates of all Gram negative isolates, or clinical data (including patient demographics, underlying conditions, and antibiotic treatment). We only included blood stream infections. The full details of inclusion and exclusion criteria are presented in Additional file 2: Table S2.

### Statistical analysis

Descriptive analysis was performed to investigate the distribution of genotype and MLST typing. Antimicrobial resistance rates were reported by median with interquartile interval (IQI).

## Results

### Literature search and study selection

**Fig. 1:**
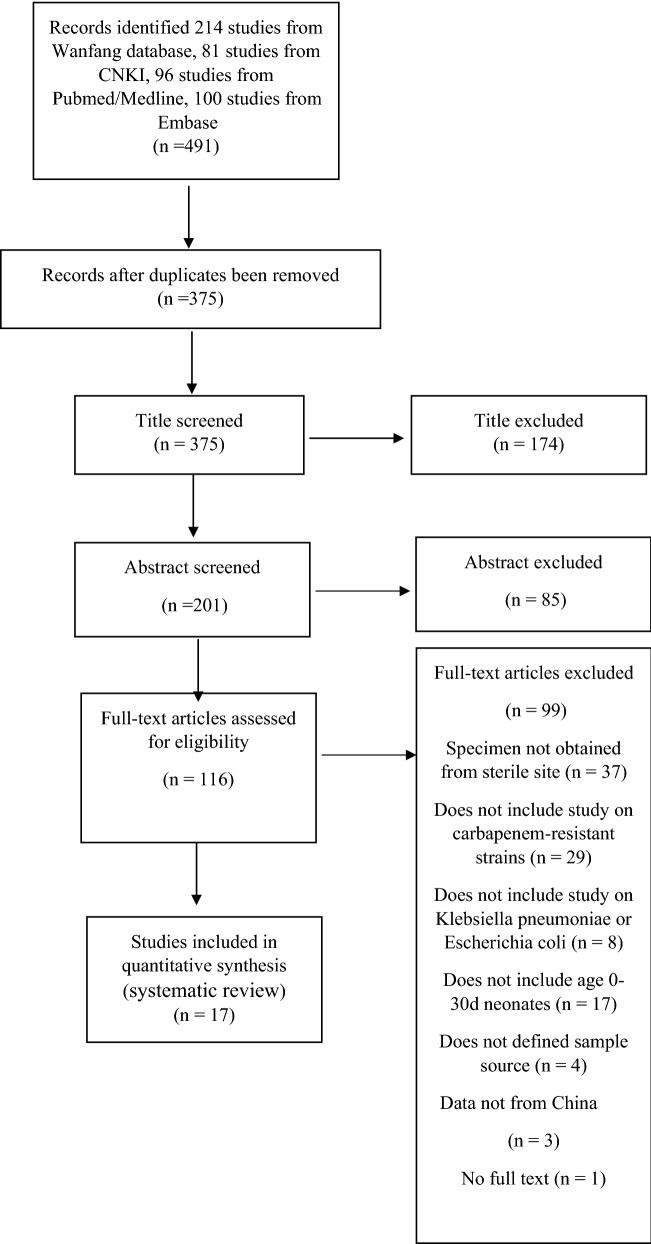
Search strategy and process of study selection

We identified 491 studies from Chinese and English database searches: 81 from CNKI, 214 from Wanfang med database, 96 from Pubmed/Medline and 100 from Embase (the flow chart is shown in Fig. 1). A total of 17 studies met the inclusion criteria and were included for final review, of these 11 (64.7%) reported genotype (including carbapenemase, β-lactamase genes and AmpC cephalosporinases genes) distribution and 9 (52.9%) reported AMR and clinical data. Only 6 studies reported treatment outcomes and gave the proportion of carbapenem-resistant isolates relative to all Gram-negative isolates. The full list of studies included in the review is available in Additional file 3: Table S3.

### Demographics and clinical presentations of *K. pneumoniae* or *E. coli* infections

All 17 studies were from tertiary hospitals. Based on the Government economic divisions of China, 7 studies were from Eastern China, 6 studies from Central China, and 4 studies from Western China. Only 9 of 17 studies reported clinical data, including patient demographics, underlying conditions, and antibiotic treatment. A total of 16 infants were included in these 9 studies. Eight of 16 patients were reported to have underlying conditions, including 6 with lung disease, 2 with necrotizing enterocolitis (NEC) and 2 with recent surgery. Ten patients in these 9 studies reported antibiotic treatment: 5 received meropenem alone, 1 ceftazidime alone, 3 patients had received two antibiotics (piperacillin/sulbactam and ceftazidime; imipenem and amikacin; meropenem and ciprofloxacin) and 1 patient had received more than three antibiotics. Eight patients in these 4 studies had received antibiotics prior to the onset of the relevant infection: 5 received meropenem, 1 received panipenem and 2 didn’t report the type the antibiotics. Clinical treatment outcomes were reported in 13 patients from 6 studies; 3 died and their deaths were attributed to the infection. Eleven patients of the six studies reported the source of infection. Ten patients (10/11, 90.9%) were hospital-acquired infections, while only one was considered to be a vertical transmission.

### The proportion of carbapenem resistant strains of all Gram negative strains

Only 6 studies (35%; 6/17) reported the proportion of carbapenem resistant isolates relative to all Gram-negative isolates causing sepsis. Overall, 39 (5.3%) carbapenem resistant *K. pneumoniae* and *E. coli* isolates were reported of out of a total of 740 Gram-negative isolates.

### Antimicrobial resistance genotype and Multilocus Sequence Type (MLST)

Genotypic data were available for 21 isolates in 11 studies (20 *K. pneumoniae,* 1 *E coli)*. The most commonly reported genotype was NDM-1 (81.0%, 17/21), followed by KPC-2 (9.5%, 2/21) and IMP-4 (9.5%, 2/21). 15 isolates from 9 studies were tested β-lactamase genes, 66.7% (10/15) isolates carried TEM and SHV genotypes, and 80.0% (12/15)carried CTX-M. 7 isolates from 5 studies were Amp C gene positive, and more than half of them were CMY-4/30 (57.1%; 4/7).

Antibiotic susceptibility results were reported from 19 isolates in 9 studies. The resistance rates of carbapenem-resistant *Klebsiella pneumoniae* (CRKP) and carbapenem-resistant *Escherichia coli* (CREC) to second-, third-, and fourth-generation cephalosporins were 100% (IQI 100%–100%). All isolates were susceptible to colistin and fosfomycin (Table 1). MLST was identified for 14 isolates (13 *K. pneumoniae* and 1 *E.coli*) from 8 studies. ST105 was the most common *K. pneumoniae* ST type (30.8%; 4/13), followed by ST17 and ST 20 with 23.1% (3/13) and 15.4% (2/13), respectively (Table 2).

**Table 1:** The proportion of isolates demonstrating antimicrobial resistance

First author	Publication year	Sample	Sample size (number)	Aztreonam %	Levofloxacin %	Ciprofloxacin %	Gentamicin %	Amikacin %	Tigecyclin %	Imipenem %	Meropenem %	Ertapenem	Cefatriaxone %
He et al.	2017	KP	5	100						100	100		100
Jiang et al.	2012	KP	1	0	0		0	0		0	100	100	
Jin et al.	2015	KP	1	100	0		0	0	0	100	100	100	
Zheng et al.	2016	KP	4	0	0	0		0	0	100	100		
Liu et al.	2013	KP	1	100	100	100	100			100			
Zhang et al.	2015	KP	3	0	0	0	0	0		100	100		100
Zhang et al.	2015	KP	1			100		100	0	100	100	100	100
Qin et al.	2014	E.coli	1	100	0	100	100	0	0	100		100	
		KP	1	100	0	0	100	0	100	100		100	
Jin et al.	2017	KP	1	100	0		0	0	0	100	100	100	100
Meidan				100	0	50	0	0	0	100	100	100	100
IQI 25%				0	0	0	0	0	0	100	100	100	100
IQI 75%				100	0	100	100	0	0	100	100	100	100

First author	Publication year	Sample	Sample size (number)	Cefotaxime %	Ceftazidim %	Cefepime %	Cefoxitin %	Fosfomycin %	Piperacillin %	PIP/TZB %	Colsitin
He et al.	2017	KP	5						100		
Jiang et al.	2012	KP	1	100	100	100	100			0	
Jin et al.	2015	KP	1	100	100	100	100	0		100	0
Zheng et al.	2016	KP	4		100		100		100	100	
Liu et al.	2013	KP	1	100	100	100					
Zhang et al.	2015	KP	3	100	100	100			100		
Zhang et al.	2015	KP	1	100	100	100	100			100	
Qin et al.	2014	E.coli	1		100	100		0		100	0
		KP	1		100	100		0		100	0
Jin et al.	2017	KP	1		100	100	100	0		100	0
Meidan				100	100	100	100	0	100	100	0
IQI 25%				100	100	100	100	0	100	100	0
IQI 75%				100	100	100	100	0	100	100	0

*E. coli, Escherichia coli*; *KP, Klebsiella pneumoniae*; PIP/TZB, piperacillin/tazobactam

**Table 2:** Distribution of antimicrobial resistance genotypes and MLSTs among carbapenem-resistant isolates

First Author	Economic division	Hospital level	Year of publication	Year data collection	Sample source	CLSI Criteria (year)	Studies type	Community acquired or hospital acquired infection	Organisms and sample size (n)	Resistance gene			MLST
										Carbapenemase (n)	β-lactamase genes	Amp C	
He JR et al.	Central China	Tertiary hospitals	2017	2016.9–2016.10	Blood	2015	Case reports	UNK	KP (n = 5)	bla NDM-1 (n = 5)	–	–	–
Jiang MJ et al.	Eastern China	Tertiary hospitals	2012	2009.7	Blood	2011	Case reports	UNK	KP (n = 1)	bla KPC-2 (n = 1)	bla CTX-M-14 (n = 1), bla SHV-2 (n = 1)	bla DHA-1 (n = 1)	–
Xu C et al.	Eastern China	Tertiary hospitals	2015	2013.4–2013.5	Blood	2013	Case reports	Hospital acquired	KP (n = 1)	bla NDM-1 (n = 1)	bla TEM-1 (n = 1),	–	ST22 (n = 1)
Yao MZ et al.	Eastern China	Tertiary hospitals	2003	1997.1–2002.8	Blood	UNK	Cross-sectional study	UNK	KP (n = 1)	–	–	–	–
Jiang *DQ* et al.	Western China	Tertiary hospitals	2017	2013.1–2016.12	Blood	UNK	Cross-sectional study	UNK	KP (n = 2)	–	–	–	–
Song HY, et al.	Eastern China	Tertiary hospitals	2012	2009.1–2010.12	Blood	UNK	Cross-sectional study	UNK	KP (n = 1)	–	–	–	–
Zhang ZM et al.	Central China	Tertiary hospitals	2014	2011–2013	Blood	UNK	Cross-sectional study	UNK	KP (n = 18) E. coli (n = 9)	–	–	–	–
Tai SH, et al.	Central China	Tertiary hospitals	2017	2014.1–2016.6	Blood	2013	Cross-sectional study	UNK	KP (n = 1)	–	–	–	–
Tian *HR,* et al.	Western China	Tertiary hospitals	2016	2013.1–2014.12	Blood	UNK	Cross-sectional study	UNK	KP (n = 7)	–	–	–	–
Chen *S* et al.	Western China	Tertiary hospitals	2014	2009.1–2010.12	Blood	2010	Cross-sectional study	Hospital acquired infection	KP (n = 1)	bla IMP-4 (n = 1)	–	–	–
Jin Y, et al.	Eastern China	Tertiary hospitals	2015	2012.8–2013.9	Blood	2013	Cross-sectional study	Hospital acquired infection	KP (n = 1)	bla NDM-1 (n = 1)	bla TEM-1 (n = 1), bla CTX-M -14 (n = 1),	bla DHA-1 (n = 1)	ST20 (n = 1)
Zheng *R,* et al.	Western China	Tertiary hospitals	2016	2014.1–2014.3	Blood	2013	Cross-sectional study	Hospital acquired infection	KP (n = 4)	bla NDM-1 (n = 4), bla IMP-4 (n = 1)	bla CTX-M-15 (n = 4), bla SHV-1 (n = 4)	–	ST105 (n = 4)
Liu Y, et al.	Eastern China	Tertiary hospitals	2013	2010.6–2010.9	Blood	2009	Cross-sectional study	UNK	KP (n = 1)	bla KPC-2 (n = 1)	bla SHV-12 (n = 1), bla TEM-1 (n = 1), bla CTX-M -14 (n = 1),	–	UD (n = 1)
Zhang XY, et al.	Central China	Tertiary hospitals	2015	2012.8–2013.3	Blood	2012	Case report	Hospital acquired infection (n = 2) vertical transmission infection (n = 1)	KP (n = 3)	bla NDM-1 (n = 3),	TEM-1 (n = 3), bla CTX-M-15 (n = 3), bla SHV-1(n = 3),	bla CMY-4 (n = 3)	ST17 (n = 3)
Zhang Y, et al.	Central China	Tertiary hospitals	2015	2013.2.18	Blood	2014	Case report	Hospital acquired infection (n = 1)	KP (n = 1)	0	SHV-11 (n = 1), TEM-53 (n = 1),	0	ST65 (n = 1)
Qin SS, et al.	Central China	Tertiary hospitals	2014	2011.6–2012.6	Blood	2012	Cross-sectional study	UNK	KP (n = 1)	bla NDM-1 (n = 1)	bla TEM-1 (n = 1), CTX-M-15(n = 1)	–	ST966 (n = 1)
									*E.coli *(n = 1)	bla NDM-1 (n = 1)	bla TEM-1(n = 1)	bla CMY-30 (n = 1)	ST40 (n = 1)
Jin Y, et al.	Eastern China	Tertiary hospitals	2017	2013.7.29	Blood	2014	Cross-sectional study	UNK	KP (n = 1)	bla NDM-1 (n = 1)	bla TEM-1(n = 1), bla CTX-M-15 (n = 1)	bla DHA-1 (n = 1)	ST20 (n = 1)

CRE, carbapenem resistant *Enterobacteriaceae; E coli*, *Escherichia coli; KP, Klebsiella pneumoniae*; NDM, New Delhi Metallo-beta-lactamase-1; Amp C, AmpC cephalosporinases; MLST, Multilocus sequence types; UD, unidentified

## Discussion

This is the first review of carbapenem-resistant *Enterobacteriaceae* (CRE) sepsis in Chinese neonates. Although the prevalence of adults and children with infections resistant to imipenem and meropenem reported by CHINET in 2017 increased significantly, the samples were mainly derived from non-sterile body fluids, and the data for children were not broken down by age. This review has demonstrated that there are very limited recent data on carbapenem resistant isolates in neonates in China. CRKP is reported more than CREC. NDM-1 was the most commonly reported carbapenemase genotype, consistent with previous reports from Asia [11], but different to reports from the United States, where KPC is the most common genotype identified in children [12]. It is worth noting that the CLSI breakpoints for carbapenem changed in 2010 and in 2015, the CDC revised the definition for CRE. In this review, 12 studies provided CLSI reference standards. Among the 12 studies, only one adopted the CLSI standard of 2015, and the others adopted the CLSI standard of before 2015.

In 2017, the World Health Organization published a list of priority pathogens in order to inform global AMR research. CRE is one of the highest priority pathogens for the development of new antibiotics [13], but there are few new antibiotics available. Cefiderocol, is a novel catechol-substituted siderophore cephalosporin with potent activity against meropenem-non susceptible *Enterobacteriaceae* [14], including metallo-β-lactamases (NDM-1, VIM, IMP). This is the most clinically advanced drug active against NDM carbapenem resistant organisms (CROs) infections [15], but no paediatric studies have yet commenced recruitment. The current standard treatment for NDM CRE infections is polymyxin based combination therapy [16]. However, polymixin E has complex pharmacokinetics requiring hydrolysis of the prodrug colistimethate sodium to colistin, making this less suitable for neonates and infants, and there are no pharmacokinetics data for polymixin B in neonates [17]. Other older, off patent drugs that have potential activity against CROs include fosfomycin and tigecycline, but again, these have no published PK data in neonates. In our study, we found that the currently reported carbapenem-resistant Enterobacteriaceae sepsis in neonate is mainly nosocomial infection. In view of the fact that there is no appropriate antibiotics to treat carbapenem resistant bacteria infection in neonates, it is very important to strengthen epidemiological surveillance, stringent standard infection control practices in healthcare settings, and to enhance the rational use of antibiotics.

## Supplementary information

**Additional file 1: Table S1.** Search terms.

**Additional file 2: Table S2.** Inclusion and exclusion criteria.

**Additional file 3: Table S3.** Characteristics of studies included and data type extracted for neonatal sepsis caused by carbapenem-resistant isolates.

## Data Availability

All the data for this paper can be found in the additional files. All data analyzed during this study are included in this published article.

## Abbreviations

*K. pneumonia*: *Klebsiella pneumonia*
*E. coli*: *Escherichia coli*
ESBL: extended-spectrum β-lactamase
KPC: *K. pneumoniae* carbapenemase
MBLs: metallo-β-lactamases
NDM: New Delhi metallo-β-lactamase
VIM: verona integrin-encoded metallo-beta-lactamases
IMP: imipenem-resistant Pseudomonas
OXAs: oxacillinases
CHINET: chinese antimicrobial resistance surveillance network
CLSI: Central Laboratory Standards Institute
MIC: minimal inhibitory concentration
MHT: Modified Hodge test
PRISMA: Preferred Reporting Items for Systematic Reviews and Meta-Analyses guidance
CNKI: China National Knowledge Infrastructure
AMR: antimicrobial resistance
NEC: necrotizing enterocolitis
CRKP: carbapenem-resistant *Klebsiella pneumonia*
CREC: carbapenem-resistant *Escherichia coli*
CRE: carbapenem-resistant *Enterobacteriaceae*
CROs: carbapenem resistant organisms
