# Supplementary material for: Systematic review of carbapenem-resistant Enterobacteriaceae causing neonatal sepsis in China
Source: Ann Clin Microbiol Antimicrob. 2019 Nov 14;18:36. doi: 10.1186/s12941-019-0334-9 (PMC6857301; doi:10.1186/s12941-019-0334-9)
Supplement: Supplementary file 1 — Additional file 1: Table S1. Search terms. [file 12941_2019_334_MOESM1_ESM.docx]

**Additional file 1: Table S1: Search terms**

| Database | Search strategy | Number of retrieved records  Limit year （January 1, 2000- June 28, 2018) |
| --- | --- | --- |
| Wanfang | ((主题：（“碳青霉烯类/碳青霉烯” ）and (主题：“耐药”）and（主题：（“婴儿/儿童/新生儿”）） | 214 |
| CNKI | (主题=“碳青霉烯类/碳青霉烯”and (主题=“耐药”）and（主题=（“婴儿/儿童/新生儿”）） | 81 |
| Pubmed  (OVID) | ((exp beta-lactamases/ or carbapenemase.mp.) or(carbapenem resistance.mp. ) or (carbapenem resistant.mp.) or ( drug resistance.mp. or exp drug resistance/) ( carbapenemase*.mp.) or((carbapenem adj1 resist*).mp.)or (MBL.mp.) or (metallo-b-lactamase.mp.) or (VIM.mp.) or (NDM.mp.) or (OXA.mp.) or (oxacillinase.mp.) or (IMP.mp.) or (KPC.mp.) or (Klebsiella pneumoniae carbapenemase.mp.) or (OmpK.mp.)) and ((exp Enterobacteriaceae/ or enterobacteriaceae.mp.) or (exp Escherichia/ or escherichia.mp. or exp Escherichia coli/) or (klebsiella.mp. or exp Klebsiella/ or exp Klebsiella pneumoniae/ )) and ((China.mp. or exp China/) or (Chinese.mp.)) and (neonate.mp. or exp Infant, Newborn/) | 96 |
| Embase  (OVID) | ((exp beta-lactamases/ or carbapenemase.mp.) or(carbapenem resistance.mp. ) or (carbapenem resistant.mp.) or ( drug resistance.mp. or exp drug resistance/) ( carbapenemase*.mp.) or((carbapenem adj1 resist*).mp.)or (MBL.mp.) or (metallo-b-lactamase.mp.) or (VIM.mp.) or (NDM.mp.) or (OXA.mp.) or (oxacillinase.mp.) or (IMP.mp.) or (KPC.mp.) or (Klebsiella pneumoniae carbapenemase.mp.) or (OmpK.mp.)) and ((exp Enterobacteriaceae/ or enterobacteriaceae.mp.) or (exp Escherichia/ or escherichia.mp. or exp Escherichia coli/) or (klebsiella.mp. or exp Klebsiella/ or exp Klebsiella pneumoniae/ )) and ((China.mp. or exp China/) or (Chinese.mp.)) and (neonate.mp. or exp Infant, Newborn/) | 100 |
| Total |  | 491 |
