# Supplementary material for: Systematic review of carbapenem-resistant Enterobacteriaceae causing neonatal sepsis in China
Source: Ann Clin Microbiol Antimicrob. 2019 Nov 14;18:36. doi: 10.1186/s12941-019-0334-9 (PMC6857301; doi:10.1186/s12941-019-0334-9)
Supplement: Supplementary file 2 — Additional file 2: Table S2. Inclusion and exclusion criteria. [file 12941_2019_334_MOESM2_ESM.docx]

**Additional file 2: Table S2: Inclusion and exclusion criteria**

|  | **Inclusion criteria** | **Exclusion criteria** |
| --- | --- | --- |
| **Population** | Invasive carbapenem-resistant *Klebsiella pneumoniae* or *Escherichia coli* aged 0-30 days at onset of infection | Patients aged > 30 days. Without invasive carbapenem-resistant *Klebsiella pneumoniae* or Escherichia coli infections |
| **Sample source** | Samples come from blood | Samples not from blood |
| **Search** | No language restrictions | Foreign language papers where it was not possible to obtain English or Chinese translation |
| **Article type** | Study reporting more recent data | Reviews. Studies from the same country reporting repeated years |
