# Supplementary material for: Systematic review of carbapenem-resistant Enterobacteriaceae causing neonatal sepsis in China
Source: Ann Clin Microbiol Antimicrob. 2019 Nov 14;18:36. doi: 10.1186/s12941-019-0334-9 (PMC6857301; doi:10.1186/s12941-019-0334-9)
Supplement: Supplementary file 3 — Additional file 3: Table S3. Characteristics of studies included and data type extracted for neonatal sepsis caused by carbapenem-resistant isolates. [file 12941_2019_334_MOESM3_ESM.docx]

| **First author** | **Economic division** | **Year of publication** | **Year and month of data collection** | **CLSI** | **Clinical data** | **Outcome** | **Proportion of carbapenem-resistant isolates relative to all Gram-negative isolates.** | **AMR** | **Geno-type** | **MLST** |
| --- | --- | --- | --- | --- | --- | --- | --- | --- | --- | --- |
| He J [1] | Central China | 2017 | 2016.9-2016.10 | 2015 | Y | Y |  | Y | Y |  |
| Jiang M *et al.*[2] | Eastern China | 2012 | 2009.7 | 2011 | Y |  |  | Y | Y |  |
| Xu C *et al.*[3] | Eastern China | 2015 | 2013.4-2013.5 | 2013 |  |  |  |  | Y | Y |
| Yao M *et al.*[4] | Eastern China | 2003 | 1997.1-2002.8 | Unk |  |  | Y |  |  |  |
| Jiang *D et al.*[5] | Western China | 2017 | 2013.1-2016.12 | Unk |  |  | Y |  |  |  |
| Song H, *et al.*[6] | Eastern China | 2012 | 2009.1-2010.12 | Unk |  |  | Y |  |  |  |
| Zhang Z *et al.*[7] | Central China | 2014 | 2011-2013 | Unk |  |  | Y |  |  |  |
| Tai S, *et al.*[8] | Central China | 2017 | 2014.1-2016.6 | 2013 |  |  | Y |  |  |  |
| Tian *H, et al.*[9] | Western China | 2016 | 2013.1-2014.12 | Unk |  |  | Y |  |  |  |
| Chen *S et al.*[10] | Western China | 2014 | 2009.1-2010.12 | 2010 | Y |  |  |  | Y |  |
| Jin Y, *et al.*[11] | Eastern China | 2015 | 2012.8-2013.9 | 2013 | Y | Y |  | Y | Y | Y |
| Zheng *R, et al.*[12] | Western China | 2016 | 2014.1-2014.3 | 2013 |  |  |  | Y | Y | Y |
| Liu Y, et al. [13] | Eastern China | 2013 | 2010.6-2010.9 | 2009 | Y | Y |  | Y | Y | Y |
| Zhang X, et al. [14] | Central China | 2015 | 2012.8-2013.3 | 2012 | Y | Y |  | Y | Y | Y |
| Zhang Y, *et al.*[15*]* | Central China | 2015 | 2013.2.18 | 2014 | Y | Y |  | Y | Y | Y |
| Qin S, *et al.* [16] | Central China | 2014 | 2011.6-2012.6 | 2012 | Y | Y |  | Y | Y | Y |
| Jin Y, *et al.*[17] | Eastern China | 2017 | 2013.7.29 | 2014 | Y |  |  | Y | Y | Y |
|  |  |  |  |  |  |  |  |  |  |  |

**Additional file 3: Table S3: Characteristics of studies included and data type extracted for neonatal sepsis caused by carbapenem-resistant isolates.**

Abbreviation: CLSI: Clinical and Laboratory Standards Institute criteria; AMR：antimicrobial resistance; MLST: Multilocus Sequence Type；Unk: Unknown.

**References**

1. He J. Bloodstream infection of carbapenem resistant Klebsiella pneumoniae in neonates of 5 cases. *chinese community doctors*. **2017**;32(33):20-21.

2. Jiang M, Qin X. Investigation on β-lactam resistance gene of Klebsiella pneumoniae with decreased sensitivity to carbapenem antibiotics. *Chin J Exp Clin Infect Dis*. **2012**;6(04):327-332.

3. Xu C, Liu Y, Zhao H, et al. Newborn nosocomial infection caused by New Delhi metallo-beta-lactamase-1(NDM-I)-producing Klebsiella pneumoniae ST22 isolates. *Clin J Clin Lab Sci*. **2015**;11(33).

4. Yao M, Chen C, Zhang X. Antibiotic sensitivity test and clinical analysis of 76 cases of neonatal sepsis. *Chin J Pract Pediatr*. **2003**;11(18).

5. Jiang D, Chen Q, Peng M. Pathogenic bacteria distribution and drug sensitivity analysis of 1 55 neonates with bloodstream nfection. *Med Lab Sci Clin*. **2017**;10(28).

6. Song H, Cheng X, Zhang W. Analysis of the pathogens isolated from blood specimens of neonates and their antibiotic resistance. *Lab Med*. **2012**;7(27).

7. Zhang Z, Qiu W. Bacterial flora distribution and drug resistance analysis of main pathogens in neonates after blood culture. *Maternal Child Health Care of China*. **2014**;23(29).

8. Tai S, Wang P, Shao Y, et al. Drug Resistance Analysis and Distribution Characteristics of Pathogens in Neonatal Septicemia. *Experimental Lab Med*. **2017**;4(35).

9. Tian H, Zhao B, Sun G. AnaIysis on pathogen distribution of n∞nataI bIood culture and analysis on drug Susceptibility test results. *Lab Med Clin*. **2016**;10(13).

10. Chen S, Feng W, Chen J, et al. Spread of carbapenemase-producing enterobacteria in a southwest hospital in China. *Annals of Clinical Microbiology & Antimicrobials*. **2014**;13(42):12.

11. Jin Y, Shao C, Li J, et al. Outbreak of multidrug resistant NDM-1-producing Klebsiella pneumoniae from a neonatal unit in Shandong Province, China. *PLoS ONE [Electronic Resource]*. **2015**;10(3).

12. Zheng R, Zhang Q, Guo Y, et al. Outbreak of plasmid-mediated NDM-1-producing Klebsiella pneumoniae ST105 among neonatal patients in Yunnan, China. *Annals of Clinical Microbiology & Antimicrobials*. **2016**;15(10):19.

13. Liu Y, Li XY, Wan LG, et al. Acquisition of carbapenem resistance in multiresistant Klebsiella pneumoniae isolates of sequence type 11 at a university hospital in China. *Diagnostic Microbiology & Infectious Disease*. **2013**;76(2):241-243.

14. Zhang X, Li X, Wang M, et al. Outbreak of NDM-1-producing Klebsiella pneumoniae causing neonatal infection in a teaching hospital in mainland China. *Antimicrobial Agents & Chemotherapy*. **4349**;59(7):4349-4351.

15. Zhang Y, Zeng J, Liu W, et al. Emergence of a hypervirulent carbapenem-resistant Klebsiella pneumoniae isolate from clinical infections in China. *Journal of Infection*. **2015**;71(5):553-560. PubMed PMID: 605899190.

16. Qin S, Fu Y, Zhang Q, et al. High incidence and endemic spread of NDM-1-positive Enterobacteriaceae in Henan Province, China. *Antimicrobial Agents and Chemotherapy*. **2014**;58(8):4275-4282. PubMed PMID: 373681141.

17. Jin Y, Song X, Liu Y, et al. Characteristics of carbapenemase-producing Klebsiella pneumoniae as a cause of neonatal infection in Shandong, China. *Experimental and Therapeutic Medicine*. **2017**;13(3):1117-1126. PubMed PMID: 614329576.
